# Stump refashioning technique in lower limb osseointegration

**DOI:** 10.1007/s00423-025-03961-9

**Published:** 2026-01-07

**Authors:** Muhammad Taqi, Munjed Al Muderis, Mustafa Alttahir, Kevin Tetsworth

**Affiliations:** Macquarie University Hospital, Sydney, Australia

**Keywords:** Osseointegration stump refashioning, Stump soft tissue management, Reshaping osseo-stump, Osseo-stump debridement, Soft tissue residuum stewardship

## Abstract

**Background:**

Osseointegration represents an innovative technique within the field of limb amputation. The management of a permanent stoma through the residuum presents significant challenges, primarily due to the insufficient literature and limited surgical techniques available. This study aims to unravel soft tissue management strategies that seek to enhance surgical outcomes.

**Methods and results:**

It is a retrospective study containing a total of 406 patients (251transfemoral and 155 transtibial) amputees who underwent (264 trans-femoral and 177 transtibial) osseointegration at Macquarie University Hospital and Norwest Private Hospital were systematically evaluated over the period spanning from December 2010 to December 2023. Out of a total of 264 transfemoral cases, 87(32.9%) cases necessitated stump refashioning surgery. In transtibial osseointegration, 37/177 cases (20.9%) of stump refashioning events were observed among the cohort.

**Conclusion:**

This study describes the surgical technique and the importance of careful soft tissue management in stump refashioning. It addresses issues of stoma pain, overhanging soft tissue, and infections to prevent potential complications and improve quality of life.

## Introduction

Lower limb amputations lead to significant challenges that profoundly impact the quality of life, physical activity, and psychological health. [1–3] Additionally, the associated morbidity and mortality rates highlight the severity of the influence of lower limb amputations [4]. Following an amputation, patients frequently require an extended period of rehabilitation to prepare for the fitting of traditional suspended socket prostheses. Fitting becomes considerably more challenging with higher levels of amputation, stump size fluctuations, and unhealthy scarred or sensitive skin and often leads to possible complications, including localized pain, excoriation, blisters, folliculitis, skin ulceration, pistoning, rotational instability, and inefficient walking due to lack of proprioception [5–7]. 

Patients who have undergone bilateral above-knee amputations often experience significant mobility limitations. Statistics indicate that more than 90% of these individuals eventually become reliant on wheelchairs due to the considerable challenges associated with using prostheses on both lower limbs [8]. Osseointegration has revolutionized the reconstruction of limb loss by eliminating most of the problems related to the traditional socket interface. During the surgical procedure, the prosthesis is directly attached to the skeletal residuum. Osseointegration includes a notable increase in the prosthesis’s stability, preservation of proprioception, elevated functional activity levels, and swift and secure attachment [9]. 

Osseointegration presents its own challenges, including skin irritation due to overhanging soft tissue, formation of granulation tissue, and localized bacterial colonization of the stoma site, all of which require ongoing treatment and potential stump refashioning. In a study by Almuderis et al., it was found that 6 out of 22 patients (27%) required elective surgery for soft tissue refashioning [10]. 

Over the last twenty years, there has been a significant evolution in surgical techniques, with a transition from two-stage to single-stage procedures, as well as advancements in soft tissue management. Soft tissue management plays a crucial role in addressing potential complications and minimizing the risk of future adverse outcomes. The aim of this study is to explore the strategies and techniques of soft tissue management in stump refashioning, with a focus on optimizing outcomes for patients undergoing osseointegration procedures. This study strives to improve the long-term success and functionality of osseointegrated prostheses for lower limb amputees by tackling potential complications and refining surgical practices.

## Materials and methods

This retrospective study was conducted after the approval of the ethical board review. Stump refashioning surgery was performed in patients presenting with localized infection, excessive granulation tissue, or irritation due to overhanging soft tissue. In the present study, a total of 441 patients (264 transfemoral and 177 transtibial) amputees who underwent osseointegration at Macquarie University Hospital and Norwest Private Hospital were systematically evaluated over the period spanning from December 2010 to December 2023. The international patients were excluded from the study because of their infrequent follow-ups. Comparative analyses using chi-square tests and independent t-tests were utilized to analyze data. Follow-up clinical assessments of these patients were done at 2 weeks, 6 weeks, 3 months, and 6 months. All patients progressed to stable mature stoma formation and returned to their pre-surgery activity levels.

## Results

264 transfemoral osseointegration procedures were performed in 251 patients; 11(4.4%) patients underwent bilateral procedures, and 241 (95.6%) patients had unilateral procedures. The average age at the time of surgery was 51.57 ± 14.27 years. The cohort comprised 194 (77%) males and 58(23%) females. The cause of amputation was trauma in 164 cases, Cancer in 30, vascular dysfunction in 13, Infection in 42, and 14 cases included other causes (Deformity, CRPS, Spinal surgery). In the transfemoral osseointegration cohort, stump refashioning surgeries were performed in 87 (32.9%) cases. Further analysis revealed 35(41.1%) cases requiring revision procedures, 16 cases undergoing three surgeries, 3 cases receiving four surgeries, and one case necessitating five procedures. The average time between refashioning surgeries revealed a mean interval of 2.54 years, with a standard deviation of 2.04 years.

177 transtibial osseointegration procedures were performed in 155 patients, including 111 male (72%) and 45(28%) female patients. The average age of participants was 52.9 ± 14.4 years. 12/155 (7.7%) patients underwent bilateral and 144 (92.3%) patients unilateral transtibial osseointegration. The cause of amputation was trauma in 88 cases, vascular dysfunction in 19, Infection in 26, cancers in 2 cases, and 21 patients had other reasons (Deformity, CRPS, Spinal surgery).

In transtibial osseointegration procedures, a total of 37/177 patients (20.9%) experienced stump refashioning events. Moreover, 9 cases required 2 refashioning events. The average time taken for refashioning across all patients was 3.36 years, with a standard deviation of 2.22 years. (Table 1)

**Table 1:** Comparison of transfemoral and transtibial osseointegration cohorts

Parameter	Transfemoral Osseointegration	Transtibial Osseointegration
Total Procedures (n)	264	177
Total Patients (n)	252	156
Bilateral Cases	11 (4.4%)	12 (7.7%)
Unilateral Cases	241 (95.6%)	144 (92.3%)
Mean Age (years)	51.57 ± 14.27	52.9 ± 14.4
Sex (Male : Female)	194 (77%) : 58 (23%)	111 (72%) : 45 (28%)
Cause of Amputation	Trauma: 165	Trauma: 88
	Cancer: 30	Cancer: 2
	Vascular: 13	Vascular: 19
	Infection: 42	Infection: 26
	Other (Deformity, CRPS, Spinal): 14	Other (Deformity, CRPS, Spinal): 21
Stump Refashioning Surgeries	87/264 (32.9%)	37/177 (20.9%)
Multiple Refashioning Events	35 (41.1%) required revision	9 cases required 2 refashioning events
	16 had 3 surgeries	
	3 had 4 surgeries	
	1 had 5 surgeries	
Mean Interval Between Refashioning (years)	2.54 ± 2.04	3.36 ± 2.22

## Soft tissue management

### Preoperative assessment

When examining the residual limb, it is crucial to carefully assess the condition of the skin, the presence of scars, and any overhanging soft tissue. Overhanging soft tissue can lead to problems such as excessive discharge from the stump, recurrent infections, and discomfort from an implant. Additionally, friction between metal and skin can cause impressions or abrasions on the skin. Skin that appears red, swollen, and shiny may be an indication of a stump infection, which might require further workup and treatment with antibiotics or, in cases of recurrent or resistant infections, potential refashioning of the stump. The radiographs are an initial assessment tool for infected stumps to check for the presence of distal bone osteolysis. (Table 2)

**Table 2:** Indications for stump refashioning in lower limb osseointegration

Soft-tissue
Loose skin causing abrasion/ulceration from implant contact
Excessive redundant soft tissue can cause end-of-day aching pain due to the accumulation of interstitial fluid
Stoma site infection (erythema, swelling, purulent discharge)
Excessive granulation tissue resistant to local measures (e.g., silver nitrate)
Neurological
Symptomatic neuroma confirmed clinically/MRI
Need for revision nerve interface surgery e.g. Regenerative Peripheral Nerve Interface/Targeted Muscle Reinnervation (RPNI/TMR) due to recurrent neuroma
Bony
Localized osteomyelitis
Chronic sinus formation
Localized implant loosening (<50% bone–implant interface, without mechanical symptoms)
Loosening >50% with mechanical symptoms (start-up pain/pain on loading) may require major Osseointegration revision rather than refashioning. Surgical planning is based on symptom severity, functional limitation, and risk of progression.

It is of utmost importance to meticulously care for the stoma and conduct a thorough assessment of its shape and condition, whether it is completely sealed or wide open. A wide stoma can lead to persistent discharge and foul odors, which impact the patient’s quality of life. In addition, a comprehensive examination of the extent and size of any granulation is essential. This may necessitate the application of silver nitrate or the consideration of a surgical excision during the process of stump refashioning. Furthermore, the presence of a sinus around the stoma is indicative of a chronic bone infection, particularly in the externalized part of the bone. To gain deeper insights into this condition, a CT scan was employed to trace the sinus extending into the bone. It is essential to recognize that addressing the sinus will likely be a critical component of the stump refashioning procedure in order to ensure the patient’s long-term well-being.

Pain elicited by tapping nerve endings can be an indicator of potential neuroma formation. Neuropathic pain typically presents as shooting, sharp pain in the specific distribution of the affected nerve. To further assess neuropathic pain, MRI imaging or localized diagnostic injections are often utilized for more detailed investigation and diagnosis.

In patients with mild distal osteomyelitis but no functional limitation or active complaints, a strategy of watchful monitoring and regular follow-up is adopted. If infection progresses, stump refashioning becomes warranted. Symptomatic infections that fail to respond to empirical oral antibiotics generally require surgical intervention. Similarly, granulation tissue or redundant soft tissue may remain asymptomatic; therefore, management is individualized according to the severity of the problem and its impact on patient function.

### Surgical technique

The patient’s position depends upon the site of overhanging soft tissue and the type of nerve that needs to be addressed. The preferred position is supine, with a sandbag under the pelvis, which gives some access to the back of the leg as well. The use of a tourniquet is not obligatory. In the event that a tourniquet is employed, it should be at a sufficient distance from the stoma to ensure that it does not interfere with soft tissue balance. It is imperative that tourniquets are released subsequent to the ligation of principal vessels to ensure sufficient hemostasis prior to the closure of soft tissue.

The skin is scrubbed with chlorhexidine, especially around the implant and stoma. Standard prepping and draping are performed according to protocol, ensuring adequate exposure. The external component of osseointegration is wrapped with a sterile light handle or Ioban to minimize contamination of the surgical area.

An elliptical skin incision is made around a stoma that includes old scars or sinuses. The incision is skewed towards hanging soft tissue with the aim of debulking. The incision should be deep enough to raise the fish mouth fasciocutaneous flaps. The excess subcutaneous fat is debrided down to muscle without compromising the circulation to the overlying skin.

Muscles are mobilized carefully to expose the distal end of the bone without violating the periosteum. The periosteum should be respected to minimize bone necrosis, which serves as a nidus for colonization. Scarred tissue is excised till bleeding healthy tissues. Unnecessary dissection through muscles is discouraged as it may cause further damage to healed structures.

**Fig. 1:**
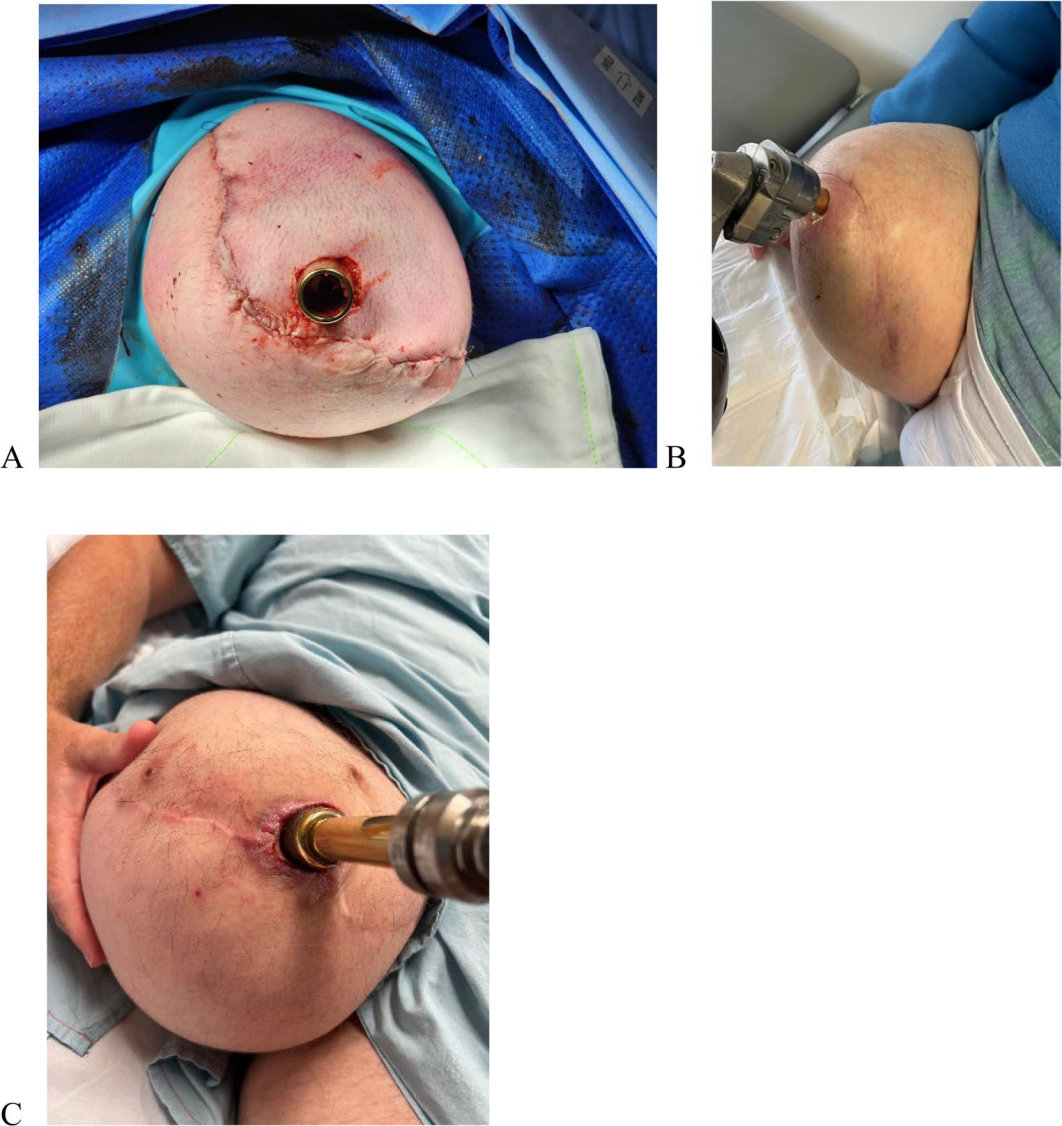
**A** Stoma recreation through the anterior flap, **B** Stoma through the posterior flap, **C** Stoma through fish mouth flap closure

The bone implant interface is explored to evaluate the distal bone-implant integration. The biofilm between the implant and the bone is debrided using a fine dental pick or needles. Pulse lavage is done to clear the dead slough present at the interface. If the cavity is large, then it is de-roofed for adequate debridement using bone nibblers or fine small osteotomes. The devascularized bone is excised to a bleeding healthy segment of bone. The cavity is filled with antibiotic-mixed bone stimulan or cerament, which helps in bone induction (Fig. 1).

The planned neuroma excision is explored through muscle planes, and then RPNI (regenerative peripheral nerve interface) or TMR(targeted muscle reinnervation) nerve procedures are undertaken. Thin nerves (saphenous, sural, lateral cutaneous femoral nerve) are dealt through TMR procedures, which involves connecting the transected nerve to a nearby motor nerve. The nerve transfer is then enveloped into a biocompatible porcine-derived extracellular matrix nerve wrap, preventing escape neuroma formation. In relatively bigger nerves(sciatic nerve), RPNI is preferred to prevent size mismatch between nerves. This process involves wrapping the nerve end into a piece of muscle using a nonabsorbable fine suture. The size of the muscle is approximately 2–3 cm in size as it relies on the graft’s revascularization from both the nerve’s intrinsic blood supply and the surrounding wound bed.

**Fig. 2:**
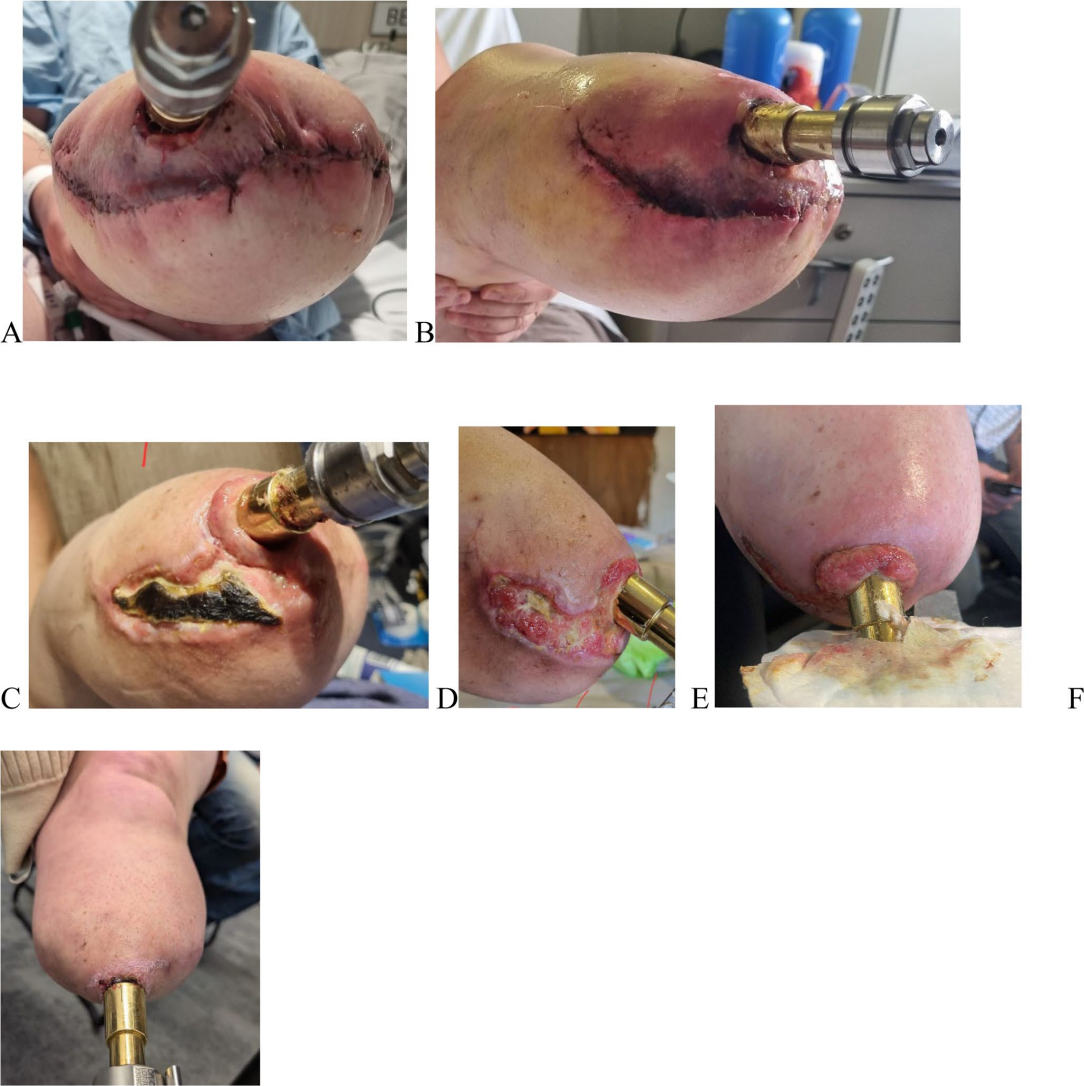
Soft tissue infection managed with antibiotics 3 months after surgery **A**-**E**, Wound approximated through secondary intention. Granulation tissue excised by local excision as shown in Figure F

**Fig. 3:**
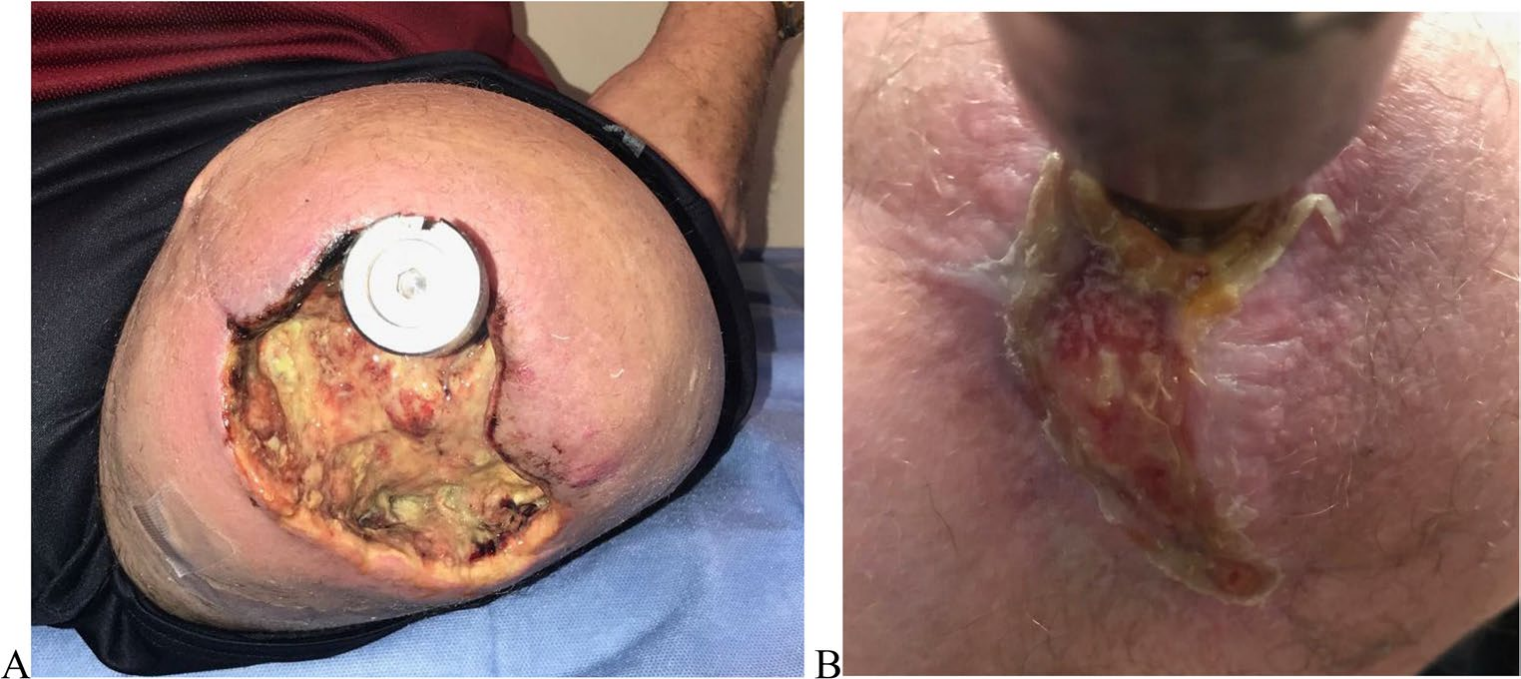
Flap Necrosis healing through secondary intention

The granulation tissue is excised completely from the base, and the underlying feeding vessels are cauterized. Adequate closure and skin approximation prevent its recurrence (Figs. 2 and 3).

**Fig. 4:**
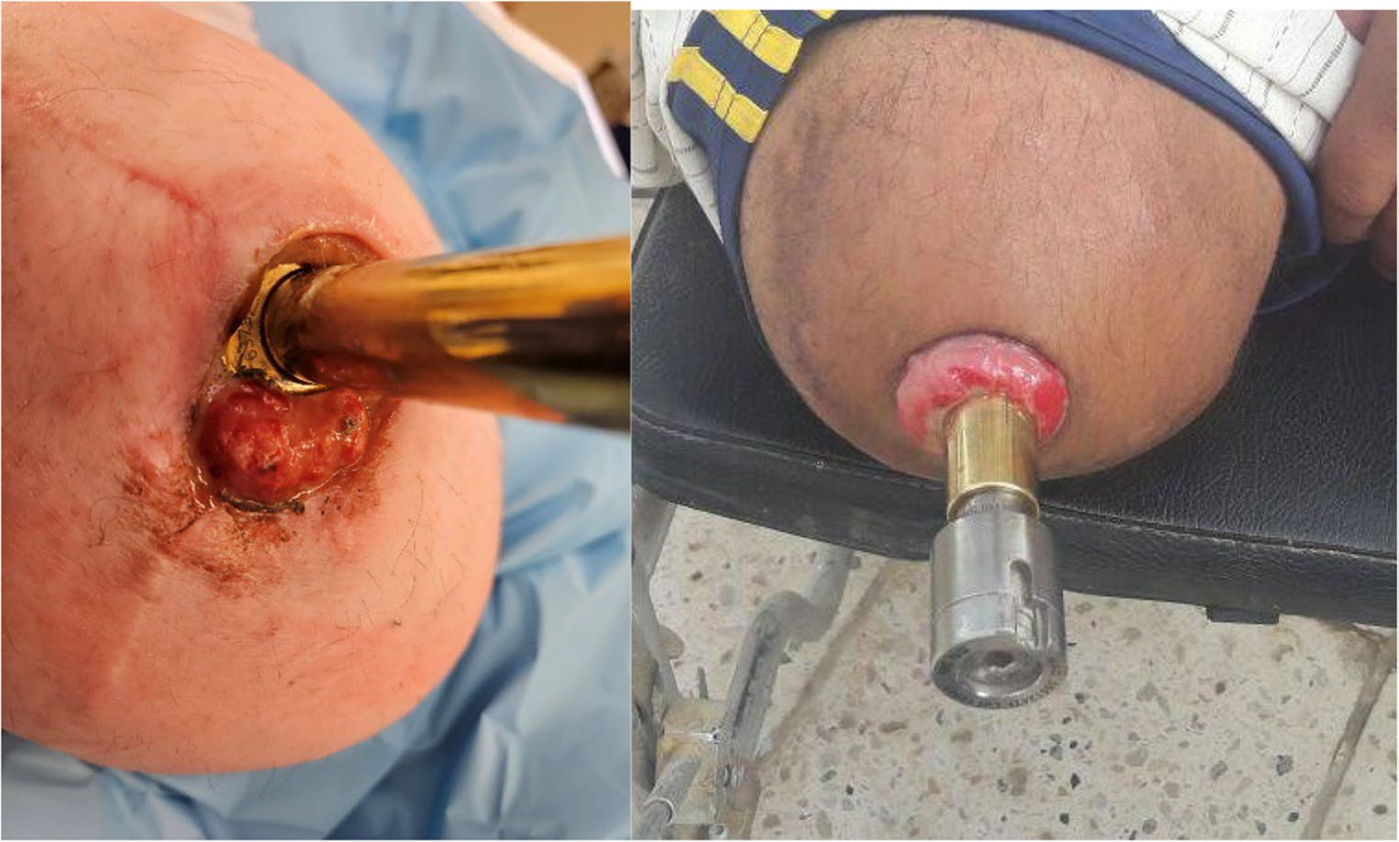
Granulation Tissue

After dissection, the surgeon performs hemostasis to control bleeding from the soft tissues. This may involve the use of manual pressure, electrocautery, ligatures, or other hemostatic agents. (Figures 2 and 4)

Muscles are stitched back to the periosteum, securing around the distal end of the bone, followed by deep fascia of muscles. The flexors are reattached to the extensors using their fascia and around the collar of the bone to create a proper myodesis around the distal end of the bone. After fascial closure, the lower skin flap is secured with an upper flap under gentle tension, leaving a lip of extra skin, which is later debrided. As the posterior flap is advanced, progressive tension sutures can be strategically placed to reduce tension along the incision line and minimize the presence of dead space. Two cutaneous sutures are placed to approximate it around the implant. The closure should not be under too much tension to prevent wound necrosis and potential wound dehiscence. If a longer anterior or posterior skin flap is available, an apple core device is used to make an opening, allowing the implant to protrude away from the surgical wound. (Fig. 1) It is common for the stoma to widen up immediately after surgery. The stoma shrinks over the following weeks and matures in months.

The wound is dressed and followed weekly to assess any sign of infection. The patient remains on a prolonged antibiotic course to prevent the possibility of infection, as the flora of the normal stoma has been disturbed by surgical intervention. The antibiotic is targeted against the isolated bacteria identified on culture sensitivity. Suture removal is delayed (3–4 weeks) to prevent any wound dehiscence or widening of the stoma.

After stump refashioning, soft tissue flap necrosis, stoma dehiscence, stoma infection, and a variable amount of discharge are possible complications. Close monitoring, wound dressings, and antibiotics are the mainstay of treatment. (Fig. 2)

In the event of necrosis, the senior author recommends refraining from interventions and allowing the wound to heal naturally through secondary intention. Additionally, regular assessment and monitoring are necessary until the wound edges come together. (Fig. 3)

Antibiotic-loaded Cerament™ was used selectively when extensive bone debridement was necessary for osteomyelitis or peri-implant bone loss, particularly in the presence of dead space requiring obliteration. Debridement followed established osteomyelitis principles—removing all infected/necrotic tissue to healthy bleeding bone. Cerament™, containing hydroxyapatite and calcium sulfate, served both as an osteoconductive scaffold for long-term bone formation and as a carrier for localized antibiotic delivery, thus reducing infection risk. The material was gradually replaced by bone over time. We observed no adverse effects, such as chronic drainage, a foreign-body response, or accelerated loosening, during the follow-up period. The extent of its long-term efficacy will be presented in a future dedicated manuscript.

## Discussion

Patients with osseointegration have some ongoing challenges that need to be rectified down the line of management. Stump refashioning is a frequently performed surgical procedure for overhanging soft tissue, frictional skin rub, recurrent localized skin infection, localized bony colonization/radiolucency, granulation tissue, and possible neuroma excision. The technique has evolved over many years of experience, which tends to address all relative potential problems and minimize the recurrence and progression of underlying pathology by reducing trauma to bone and soft tissues. The meticulous handling of soft tissue and appropriate closure guarantees suitable results.

Excessive granulation tissue can form during the proliferative stage of wound healing, but it may also develop at any stage due to friction or irritation between the soft tissue and implant [11]. Maturation and epithelialization are hampered by chronic infection or implant irritation [12, 13]. In a clinical setting, it manifests as a fleshy reddish-purple, pliable, and fragile mass of tissue that typically protrudes beyond the stoma. (Fig. 2) A polished distal portion of the implant reduces friction with soft tissue, and effective infection management is essential to avoid the formation of granulation tissue.

Silver nitrate is the primary treatment for hyper-granulation tissue due to its affordability and bleeding cauterization properties [14]. Other options include polyurethane foam dressings for moist wound healing and pulsed-dye laser therapy targeting hemoglobin [11, 15]. Additional alternative therapies, such as imiquimod, papase, salt baths, liquid nitrogen cryotherapy, and antimicrobial dressings with silver or iodine, can also be effective in certain situations [16–18]. 

Surgical debulking is frequently performed on patients who exhibit more pronounced hyper-granulation and who do not respond well to conservative management [19]. Stoma hypergranulation was reported in seventeen out of 86(19.7%) atients by Almuderis et al. [20] According to D. Reetz, hypergranulation occurred in 20.5 (8/39) of the instances. The primary treatment option for granulation tissue involved local excision or application of silver nitrate [21]. 

In the context of osseointegration, the focus regarding the skin/socket interface with sockets is redirected to the inconvenience associated with the transcutaneous stoma [9, 10]. Infection is a prevalent issue that frequently causes pain in the residuum, affecting approximately 42% of cases following osseointegration; however, implant loosening and the subsequent necessity for implant removal are exceptionally infrequent [22, 23]. The management approach for these infections varies based on factors such as the type, severity, and extent of the infection. This surgical debridement aims to prevent further spread of the infection and promote better healing outcomes for the patient. In a retrospective study, 3 out of 5 patients (60%) who had an osseointegration following burn trauma underwent stump refashioning [24]. The most prevalent adverse event indicating surgical intervention is infection, with a frequency of 5–20% [24, 25]. This study shows stump refashioning in 87 (32.9%) transfemoral cases and 17 (20.9%) transtibial osseointegration cases. A reduced number of soft-tissue procedures in transtibial osseointegration might be associated with reduced subcutaneous fat and a thin muscular envelope.

Excessive soft tissue redundancy interferes with the prosthesis, causing soft tissue irritation, stump pain, excoriations, and discomfort at the end of the day due to the accumulation of interstitial fluid due to gravity. Up to 77% of patients who have undergone osseointegrated implant surgery require further treatments due to soft tissue-related problems, according to a recent study [26]. The ideal solution for soft tissue redundancy is wearing compression stockings or surgical excision of excessive skin and soft tissue. 30 soft-tissue refashioning operations were carried out for 14 (36%) patients because of soft-tissue discomfort by D.reetz et al. [21]. Almuderis et al. reported that soft-tissue redundancy occurred in 14 cases (16%) [27]. In another study by Almuderis et al.,10 out of 50 patients required stump refashioning due to soft tissue irritation and recurrent soft tissue infections [23]. 

This study, including patients over 13 years, signifies the critical role of the stump refashioning technique in ensuring the optimal function of prosthetics. Additionally, it emphasizes the significance of timely intervention and proactive measures in preserving prosthetic function. By minimizing the requirement for external soft tissue support, the reduction of skin and adipose tissue by refashioning seeks to lower the amount of soft-tissue motion present in the residual limb. Stump refashioning also reduces the risk of soft-tissue infection and granulation tissue, as overhanging tissue creates a moist, enclosed space that provides an ideal environment for bacterial overgrowth [28, 29]. 

Despite these issues, surgeons must go above and beyond to overcome these obstacles. As we look to the future, the process of providing this osseointegration care is expected to become more streamlined due to successful outcomes resulting from evidence-based practice. The limitations of this study include the lack of subsequent analysis of patients based on their comorbidities and risk factors.

## Conclusion

In conclusion, the evolution of surgical techniques over the past two decades, particularly the shift from two-stage to single-stage procedures and advancements in soft tissue management, has significantly enhanced the outcomes of osseointegration for lower limb amputees. This study affirms the importance of meticulous soft tissue management in stump refashioning to address potential challenges such as overhanging soft tissue, localized infections, and granulation tissue formation. The findings highlight the prevalence of stump refashioning surgeries, the challenges in managing soft tissue complications, and the effectiveness of refined surgical techniques in reducing the recurrence of issues and improving prosthetic functionality. By optimizing preoperative assessment, surgical practices, and postoperative care, the long-term success and functionality of osseointegrated prostheses can be significantly improved, ultimately enhancing the quality of life for amputees.

## Data Availability

The datasets used and/or analyzed during the current study are available from the corresponding author on reasonable request.
